# Replacement of Missing Anterior Teeth in a Patient with Temporomandibular Disorder

**DOI:** 10.1155/2014/393627

**Published:** 2014-03-04

**Authors:** Satheesh B. Haralur, Omar Saeed Al-Shahrani

**Affiliations:** ^1^Department of Prosthodontics, College of Dentistry, King Khalid University, Abha 61417, Saudi Arabia; ^2^College Of Dentistry, King Khalid University, Saudi Arabia

## Abstract

The loss of anterior teeth leads to extreme psychological trauma, along with functional and esthetic debilitations. Healthy anterior teeth play an important role of protecting the posterior teeth during excursive mandibular movement. Loss of anterior teeth induces posterior interference with extended disocclusion time. Posterior disocclusion is critical to remove the harmful force on the teeth temporomandibular joint and eliminate muscle hypertonicity. Occlusal interference is considered as contributing factor to temporomandibular disorder (TMD) symptoms. Prosthesis design should eliminate deleterious tooth contacts. Establishing optimum anterior guidance is a key to establishing harmonious functional occlusion in addition to the correction of the esthetic and phonetic disabilities. This case report explains the steps involved in the rehabilitation of the TMD patient with loss of maxillary anterior teeth.

## 1. Introduction

Teeth are important to humanity not only from functional point, but also because they contribute substantially towards psychological well-being of the person. Loss of the teeth in the young adults will adversely impact the self-concept and social integration [1]. The tooth loss in younger patients is mainly attributed to the genetic, caries, and traumatic injuries. Absence of anterior teeth in addition to esthetic and phonetic handicap will affect the anterior guidance of the patients. It is critical for the clinician to understand the effect of palatal surface of the maxillary anterior teeth on mandibular movement before the initiation of their replacement [2].

Improper restoration or replacement of the anterior teeth leads to harmful interference in the posterior teeth, compromised esthetics, and mechanical failures of the prosthesis [3]. Faulty prosthesis design predisposes the supporting structures of the abutment teeth for damage [4]. Optimum anterior guidance will also help the disocclusion of posterior teeth in protrusive and lateral mandibular excursive movements [4]. Loss of anterior teeth leads to prolonged disclusion time, which may initiate temporomandibular disorders. The patient with missing anterior teeth along with existing TMD requires the careful integration of many restorative principles for successful management [5]. The anterior guidance with satisfactory esthetics, phonetics, and comfort along with optimum disocclusion time is crucial for successful rehabilitation of TMD patient [6].

This case report explains the clinical methodology in restoring missing anterior teeth in the patients with temporomandibular disorder.

## 2. Case Presentation

**Figure 1:**
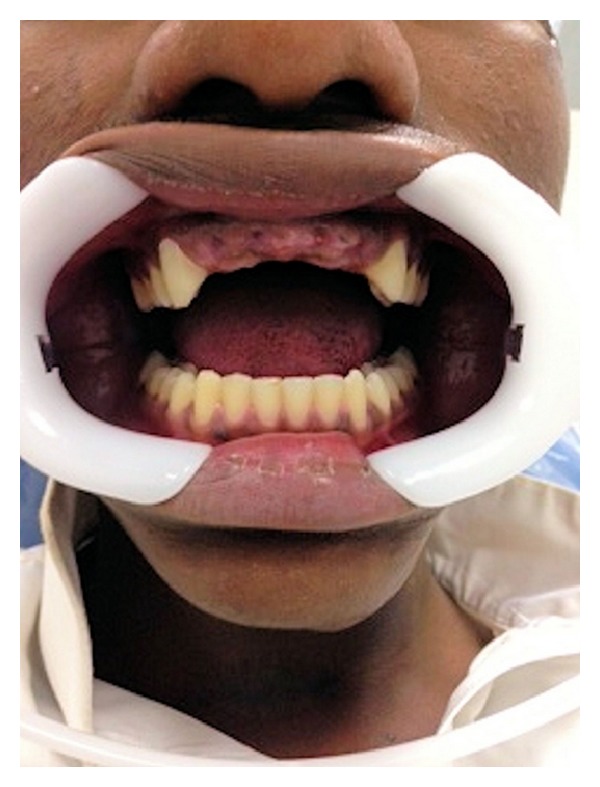
Photograph showing the missing teeth.

**Figure 2:**
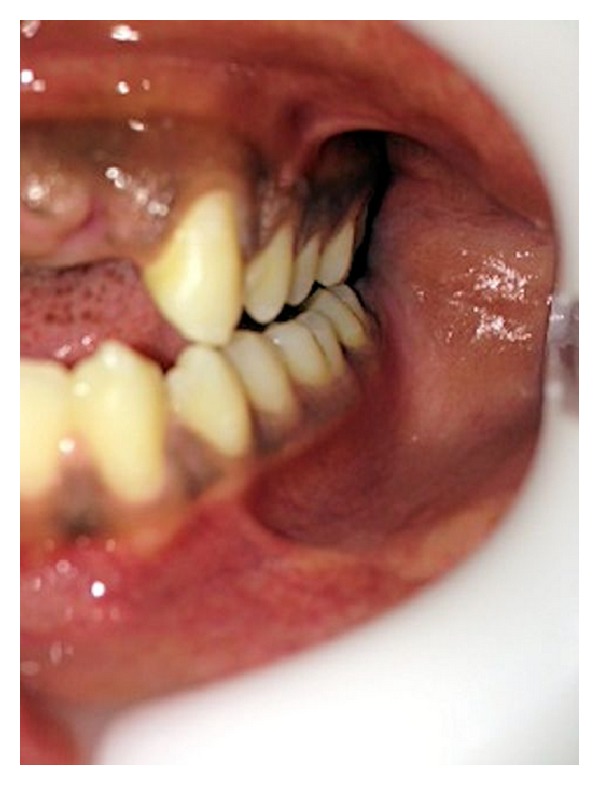
Posterior occlusal interference on mandibular lateral movement.

A 22-year-old male patient visited the King Khalid University Dental Clinic for the replacement of anterior missing teeth. He attributed the loss of teeth to road traffic accident 6 months back. He also complained of moderate pain and clicking in the left temporomandibular joint. Patient provided the history of admission in a tertiary referral hospital subsequent to accident to have treatment for mild concussion from neurophysician and also a maxillofacial surgeon. On examination it was observed that the patient had lost maxillary central and lateral incisors on both sides (Figure 1). The patient reported no change of posterior occlusion after accident. There was no mobility of adjacent teeth. Temporomandibular joint (TMJ) examination revealed that left and right lateral pterygoid muscles were tender to palpation. The maximum opening of the mouth was within normal range, with no restriction of lateral mandibular movement. The single click was observed on the left side of TMJ; no deviation or deflection of mandible occurred upon opening. Occlusion evaluation showed the protrusive and nonworking side contacts on left side (Figure 2). Digital occlusal evaluation was also performed with T Scan III; it confirmed the existence of protrusive and balancing side interference. The occlusion time recorded was within normal range (0.52 seconds). Protrusive and left lateral disocclusion time were 0.86 seconds and 1.79 seconds, respectively. The right lateral disocclusion time recorded was 0.74 seconds. The protrusive and left lateral disocclusion times were substantially prolonged. The panoramic and intraoral periapical radiographs showed nothing significantly abnormal in TMJ and adjacent teeth. On complete evaluation of clinical signs, symptoms, and clinical examination, it was diagnosed as loss of maxillary anterior teeth with the associated temporomandibular joint disorder.

The treatment objectives were to replace the missing maxillary central and lateral incisors along with eliminating existing occlusal interference to rehabilitate the TMJ. Treatment options to replace the missing anterior teeth were discussed with the patient and his parents. Though the implant supported fixed prosthesis was ideal treatment, due to socioeconomic factors, conventional tooth supported fixed partial denture treatment plan was finalized.

The bilateral canines were used as abutments for the prosthesis. Thorough clinical and radiological evaluation was done for the abutments; no pulpal, periapical, or periodontal pathology was observed. The abutments showed no pain on percussion or pathological mobility. The sulcus depth around them was within normal limits. Bilateral canines had a favourable crown root ratio, root configuration, and periodontal ligament area to support the missing four anterior teeth. Another important factor in favour of the tooth supported fixed partial denture was that, though the tooth loss was due to road traffic accident there was no associated large soft tissue or bone defect in the residual ridge of missing teeth.

**Figure 3:**
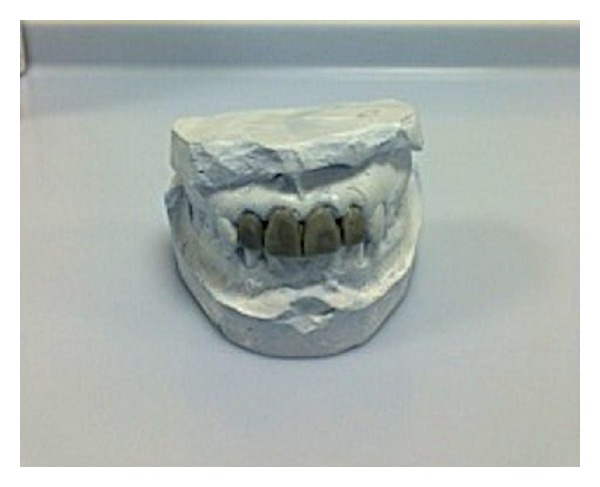
Diagnostic wax-up for the missing teeth.

**Figure 4:**
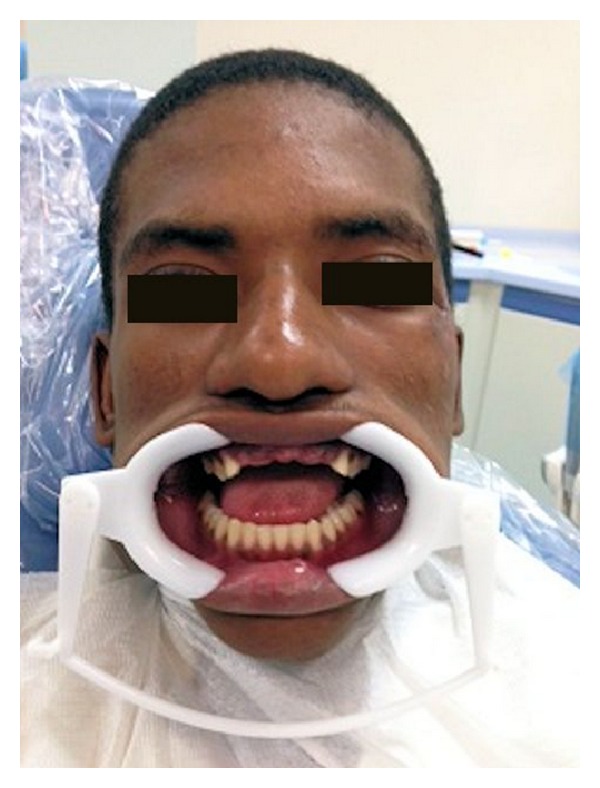
Photograph exhibiting prepared abutment teeth.

**Figure 5:**
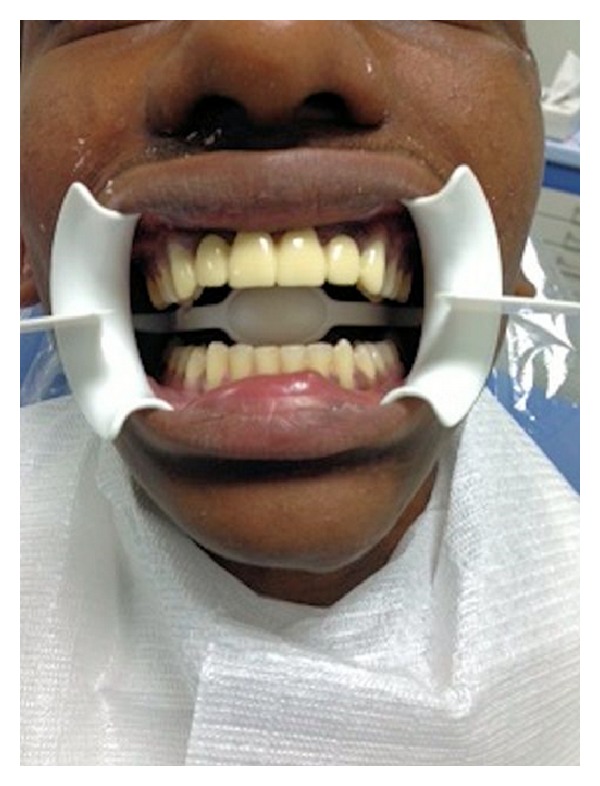
Provisional fixed partial denture in patient mouth.

**Figure 6:**
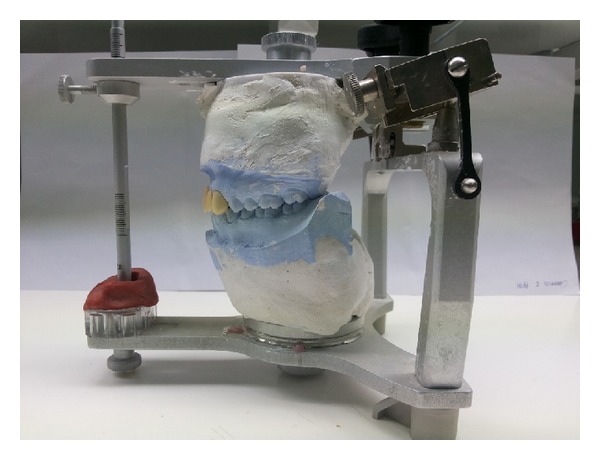
Customised incisal table made according to provisional restorations.

**Figure 7:**
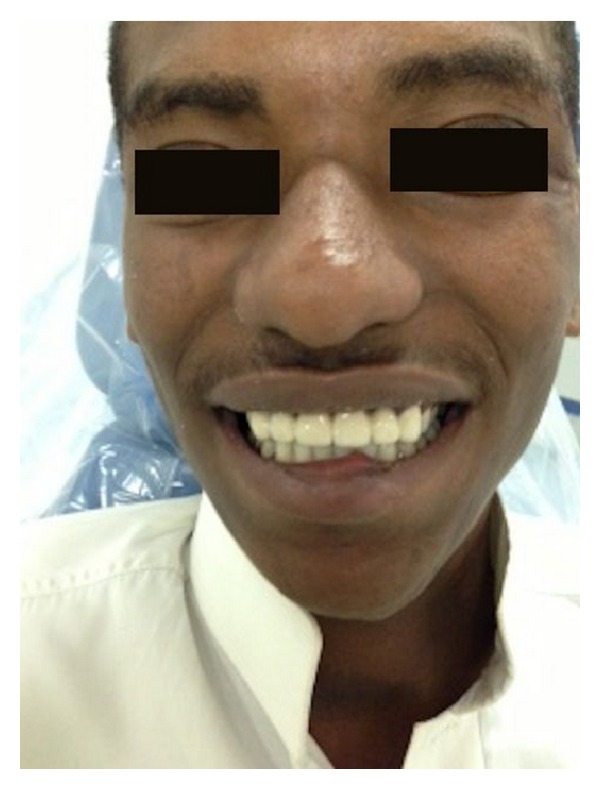
Final definitive porcelain fused with metal prosthesis in patient mouth.

Diagnostic casts were made from alginate impression, mounted on a semiadjustable articulator with the help of face bow transfer. Both abutments were prepared as full veneer porcelain fused with metal retainers (Figures 3 and 4). Provisional fixed partial denture was fabricated with indirect-direct technique. The provisional restoration was critical to evaluate esthetics and phonetics and get patient perspective on planned fixed partial denture (Figure 5). The provisional restoration was also helpful in establishing proper anterior guidance. The anterior guidance was evaluated with T Scan to adjust the protrusive disclusion time at 0.6 seconds. The occlusion refinements were made to eliminate the nonworking side contacts too by reestablishing proper canine guidance. The patient was recalled after 24 hours to evaluate the patient opinion, gingival health, and comfort. The patient was continuously monitored for 6 weeks for alleviation of pain in the TMJ. The provisional restorations with acceptable anterior guidance, esthetics, phonetics, and comfort need to be replicated in permanent restoration. The alginate impression was made with provisional restorations, poured in dental stone, and mounted on the articulator with the face bow transfer. The customized incisal guidance table was fabricated with autopolymerizing acrylic on the semiadjustable articulator (Figure 6). The working dental cast was made from additional silicone impressions of the prepared abutment teeth. It replaced the earlier dental casts in the semiadjustable articulator. Customized incisal table helped replicate the precise anterior guidance that was evaluated in the patient. The Polyvinyl siloxane putty index was made over the provisional restorations. It was helpful in making the final restoration with similar tooth contour, shape and labial surface. The final porcelain fused with metal restoration was cemented over the glass-ionomer type I luting cement after requiring occlusion refinements (Figure 7). The patient was recalled after 1 week to evaluate the residual cement, gingival health, and occlusal integrity. The patient was recalled and monitored for six months with in-between intervals of one month for prosthesis and TMJ evaluation. The patient showed complete recovery from the TMJ pain.

## 3. Discussion

Anterior tooth loss in the young patient is significantly traumatic from functional, esthetic, and psychological point. While replacing the anterior teeth, multiple factors like overbite, overjet, lip closure path, lip support, and the envelope of function should be carefully evaluated [7]. Fabricating the suitable palatal surface is as important as labial surface in anterior prosthesis. The contour and shape of the labial surface are important for esthetic purpose; the palatal surface morphology is critical for harmonious function [8].

During the anterior tooth replacement, it is better to evaluate the occlusion as a whole, since the deleterious occlusal interference can be corrected by establishing proper position, length, and overlap of the anterior teeth [9]. It is well known that occlusal interference may play a significant role in the initiation of TMD [10]. Occlusal interference may be associated with TMD centric slide, nonworking side contacts, and protrusive interference. Dynamic occlusion evaluation by T Scan showed that the occlusion and disocclusion time in TMD patients are prolonged compared to normal TMJ [11]. Hence, while restoring the anterior teeth especially in TMD patients, it is preferable to evaluate the disclusion time in protrusive and lateral side [12]. Provisional restorations are of great help for the restorative dentist to assess the esthetics and phonetics and evoke opinion from the patient and his relatives [13]. It also provides a reversible occlusal correction template to elicit the TMJ response [14, 15]. The researchers advocated many methods to transfer the acceptable anterior guidance from provisional restorations to final restoration [16, 17]. The customized incisal table is advantageous over the other method due to easy procedure and predictable results [18]. Continued, scheduled followup of the patient is required in TMD patient to monitor and introduce the required correction in later period.

## 4. Conclusion

- During anterior tooth replacement in TMD patients comprehensive occlusion evaluation is strongly advised; it will help in the elimination of deleterious tooth contacts.
- Palatal surface morphology and optimum anterior guidance are very critical to establish harmonious functions.
- Provisional restorations play an important role to evaluate not only aesthetics and phonetics but also the therapeutic effect of restoration on TMJ.
- Keep the disocclusion time shorter in protrusive and lateral mandibular movements during anterior tooth replacements.

## References

[1] Fiske J, Davis DM, Frances C, Gelbier S (1998). The emotional effects of tooth loss in edentulous people. *British Dental Journal*.

[2] Broderson SP (1978). Anterior guidance-the key to successful occlusal treatment. *The Journal of Prosthetic Dentistry*.

[3] Ogawa T, Koyano K, Suetsugu T (1997). The influence of anterior guidance and condylar guidance on mandibular protrusive movement. *Journal of Oral Rehabilitation*.

[4] Reichwage DP, Rydesky S (2004). The loss of anterior guidance as an etiological factor in periodontal pocketing. *Journal of Indiana Dental Association*.

[5] Kimmel SS (1994). Temporomandibular disorders and occlusion: an appliance to treat occlusion generated symptoms of TMD in patients presenting with deficient anterior guidance. *Cranio*.

[6] Schwartz H (1987). Anterior guidance and aesthetics in prosthodontics. *Dental Clinics of North America*.

[7] Schuyler CH (2001). The function and importance of incisal guidance in oral rehabilitation. 1963. *The Journal of Prosthetic Dentistry*.

[8] Donegan SJ, Knap FJ (1995). A study of anterior guidance. *Journal of Prosthodontics*.

[9] Hobo S (1991). Twin-tables technique for occlusal rehabilitation: part I-Mechanism of anterior guidance. *The Journal of Prosthetic Dentistry*.

[10] Marklund S, Wänman A (2010). Risk factors associated with incidence and persistence of signs and symptoms of temporomandibular disorders. *Acta Odontologica Scandinavica*.

[11] Kerstein RB (1992). Disocclusion time-reduction therapy with immediate complete anterior guidance development to treat chronic myofascial pain-dysfunction syndrome. *Quintessence International*.

[12] Kerstein RB, Wright NR (1991). Electromyographic and computer analyses of patients suffering from chronic myofascial pain-dysfunction syndrome: before and after treatment with immediate complete anterior guidance development. *The Journal of Prosthetic Dentistry*.

[13] Regish KM, Sharma D, Prithviraj DR (2011). Techniques of fabrication of provisional restoration: an overview. *International Journal of Dentistry*.

[14] Kimmel SS (1994). Temporomandibular disorders and occlusion: an appliance to treat occlusion generated symptoms of TMD in patients presenting with deficient anterior guidance. *Cranio*.

[15] Hobo S (1996). Occlusion in temporomandibular disorders: treatment after occlusal splint therapy. *International Dental Journal*.

[16] Alpert RL (1996). A method to record optimum anterior guidance for restorative dental treatment. *The Journal of Prosthetic Dentistry*.

[17] Gross MD, Cardash HS (1989). Transferring anterior occlusal guidance to the articulator. *The Journal of Prosthetic Dentistry*.

[18] Ehrlich J, Yaffe A, Hochman N (1989). Various methods in achieving anterior guidance. *The Journal of Prosthetic Dentistry*.

